# How to handle mortality when investigating length of hospital stay and time to clinical stability

**DOI:** 10.1186/1471-2288-11-144

**Published:** 2011-10-26

**Authors:** Guy N Brock, Christopher Barnes, Julio A Ramirez, John Myers

**Affiliations:** 1 Department of Bioinformatics and Biostatistics, University of Louisville, Louisville, KY 40202, USA; 2 Division of Infectious Diseases, Department of Medicine, University of Louisville, Louisville, KY 40202, USA

## Abstract

**Background:**

Hospital length of stay (LOS) and time for a patient to reach clinical stability (TCS) have increasingly become important outcomes when investigating ways in which to combat Community Acquired Pneumonia (CAP). Difficulties arise when deciding how to handle in-hospital mortality. Ad-hoc approaches that are commonly used to handle time to event outcomes with mortality can give disparate results and provide conflicting conclusions based on the same data. To ensure compatibility among studies investigating these outcomes, this type of data should be handled in a consistent and appropriate fashion.

**Methods:**

Using both simulated data and data from the international Community Acquired Pneumonia Organization (CAPO) database, we evaluate two ad-hoc approaches for handling mortality when estimating the probability of hospital discharge and clinical stability: 1) restricting analysis to those patients who lived, and 2) assigning individuals who die the "worst" outcome (right-censoring them at the longest recorded LOS or TCS). Estimated probability distributions based on these approaches are compared with right-censoring the individuals who died at time of death (the complement of the Kaplan-Meier (KM) estimator), and treating death as a competing risk (the cumulative incidence estimator). Tests for differences in probability distributions based on the four methods are also contrasted.

**Results:**

The two ad-hoc approaches give different estimates of the probability of discharge and clinical stability. Analysis restricted to patients who survived is conceptually problematic, as estimation is conditioned on events that happen *at a future time*. Estimation based on assigning those patients who died the worst outcome (longest LOS and TCS) coincides with the complement of the KM estimator based on the subdistribution hazard, which has been previously shown to be equivalent to the cumulative incidence estimator. However, in either case the time to in-hospital mortality is ignored, preventing simultaneous assessment of patient mortality in addition to LOS and/or TCS. The power to detect differences in underlying hazards of discharge between patient populations differs for test statistics based on the four approaches, and depends on the underlying hazard ratio of mortality between the patient groups.

**Conclusions:**

Treating death as a competing risk gives estimators which address the clinical questions of interest, and allows for simultaneous modelling of both in-hospital mortality and TCS / LOS. This article advocates treating mortality as a competing risk when investigating other time related outcomes.

## Background

Traditionally, mortality or survival has been the outcome of clinical interest in evaluating new ways to combat community acquired pneumonia (CAP). More recently, outcomes such as a patient's length of hospital stay (LOS) and their time to reach clinical stability (TCS) have increasingly become outcomes of interest in patients with CAP, as these are directly relevant to patient management, quality of care, and hospital costs [1-3]. Since these outcomes are time-to-event outcomes, interest lies in estimating the probability that a patient will have reached clinical stability or have been discharged from the hospital by a given day. The standard estimator for time-to-event distributions is the Kaplan-Meier estimator, and a key element of this method is the ability to handle data that are censored. For TCS and LOS, this can occur if follow-up information on a patient is unavailable after a certain point, denoted right censoring since it is only known that the event did not occur by the end of the follow-up period and it is assumed that the event of interest would have occurred at some point after this time. Another possibility is that the event of interest did not occur prior to a pre-specified time, for example when information regarding time to clinical stability is only maintained for the first week after a patient is admitted to the hospital.

A key assumption with the Kaplan-Meier estimator is that the event of interest will eventually occur for *all *patients in the population. For individuals who die prior to reaching clinical stability or being discharged from the hospital, clearly the assumption of future event occurrence in the Kaplan-Meier estimator is violated. Since an event (death) occurred to those individuals which prevents further follow-up, analysts must decide how to handle LOS and TCS data for these individuals. In the CAP literature, several ad-hoc methods for dealing with such data have surfaced: 1) disregard LOS and TCS data from individuals who die [4-6] or 2) assign the 'worst outcome' for individuals who die (i.e., right-censor them at the largest LOS or TCS value) [2,7,8]. However, it is not clear what quantity each of these ad-hoc methods is estimating, and their use can lead to differing conclusions. In addition, neither technique addresses how to estimate mortality along with LOS and TCS.

An alternative approach to handling mortality is to treat this event as a competing risk, which precludes the occurrence of the other events of interest (see [9], Chapter 8; see also [10,11]). Though these methods have been established for over thirty years, applications to outcomes in hospital epidemiology are still developing [1,12]. In the current context, when a patient dies in the hospital they can no longer reach clinical stability or be discharged from the hospital. With competing risks, probabilities of interest are the cumulative incidence functions for each event type. These functions estimate the probability of experiencing a specific event by a given time, while allowing for the possibility of other events to occur.

The goal of this article is to evaluate what the two ad-hoc approaches to handling mortality are estimating, and demonstrate the potential disparity in results which can occur from naive use of these approaches. Results from the ad-hoc estimators are further compared with the advocated way of handling mortality as a competing risk. Differences between the estimators are illustrated using data from the international Community Acquired Pneumonia Organization (CAPO) database [3], as well as simulated data. Supplemental material is also provided which gives illustrative statistical code for investigators to use in their own studies.

## Methods

### Notation and Definitions

**Figure 1:**
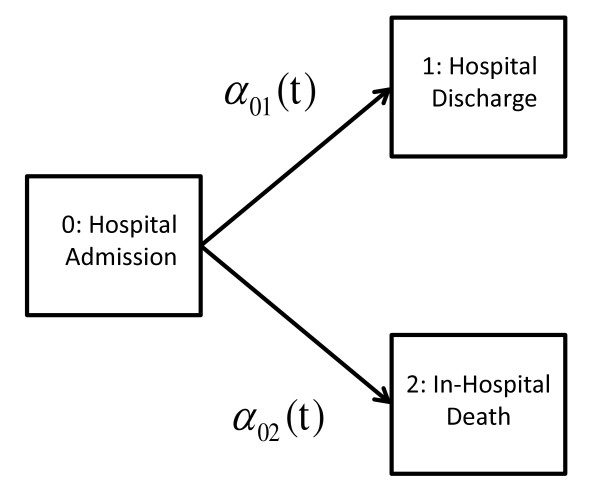
**Competing risks framework for discharge and in-hospital mortality**. Diagram illustrating the competing risks framework, with discharge and in-hospital mortality as the two competing events with transition hazards *α*_01_(*t*) and *α*_02_(*t*), respectively.

In this section, we define the functions to be estimated. The presentation given here follows in spirit to that given in Allignol *et al*. [10,13], see also [14] for a nice tutorial. Note that while we restrict attention to two competing event types, the methods extend generally to any finite number of competing events. Let (*X_t_*)_*t*≥0 _represent the state that an individial is in for every time point, *X_t _*∈ {0, 1, 2}. The initial state *X_t _*= 0 is the starting state at time 0 (hospital admission), *X_t _*= 1 represents hospital discharge or clinical stability, and *X_t _*= 2 indicates in-hospital mortality. For simplicity of presentation, we will restrict attention to hospital discharge, since the modeling of clinical stability is analogous (but see comments in Discussion). The two states discharge and in-hospital mortality represent *competing events*, since a transition into either of the two states precludes a transition into the other. In the terminology of general multi-state models, they are referred to as *terminal *or *absorbing *states. The possible transitions from hospital admission into each of them are considered *competing risks*, and the state space and possible transitions are represented schematically in Figure 1. The stochastic system is fully characterized by the two *cause-specific hazards α*_01_(*t*) and *α*_02_(*t*), which give the instantaneous rates of making making a 0 → 1 or 0 → 2 transition at time *t*, respectively. The cause-specific hazards can be thought of more intuitively using the following approximate relationship (see [10]),

(1) $$\alpha_{0j}\left(t\right)dt\approx P\left(T\in dt,X_{T}=j|T\geq t\right),\;j=1,2.$$

The quantity $\alpha_{0j}\left(t\right)dt$ then represents the probability of making a 0 → *j *transition within the infinitesimal interval *dt *= [*t*, *t *+ *dt*), where *dt *is used to represent both the interval and its length. The random variable *T *represents the transition time.

The cause-specific hazards sum to give the *all-cause hazard*, *α*_0_.(*t*)*dt *≈ *P*(*T *∈ *dt*|*T *≥ *t*), where the '·' indicates summation over the subscript. A key quantity for statistical inference is the cumulative cause-specific hazard $A_{0j}\left(t\right)=\int_{0}^{t}\alpha_{0j}\left(u\right)du$, which represents the total accumulated hazard for making a 0 → *j *transition by time *t*. Using the cumulative all-cause hazard *A*_0_. (*t*), the survival function for *T *is then a function of both cause-specific hazard functions, *S*(*t*) = *P*(*T > t*) = exp{-*A*_0_.(*t*)} [13].

When competing risks are present, probabilities are described in terms of the cause-specific *cumulative incidence functions*,

(2) $$CI_{j}\left(t\right)=P\left(T\leq t,X_{T}=j\right)=\int_{0}^{t}P\left(T>u-\right)\alpha_{0j}\left(u\right)du,j=1,2.$$

The integrand on the right-hand side of the equation represents the joint probability of having survived (i.e., made neither transition) to time just prior to *u *(denoted *u*-) and subsequently making a 0 → *j *transition at time *u*. Like the cause-specific hazard functions, the two cumulative incidence functions can be summed, resulting in the all-cause distribution function *P*(*T *≤ *t*) = *CI*_1_(*t*) + *CI*_2_(*t*).

In addition to the cause-specific hazards, another hazard function which has been developed in the competing risks literature is the *subdistribution *hazard [15,16]. This hazard function is obtained from the cumulative incidence function,

(3) $$\lambda_{j}\left(t\right)=\frac{dCI_{j}\left(t\right)∕dt}{1-CI_{j}\left(t\right)}=\frac{-d\log\left\{1-CI_{j}\left(t\right)\right\}}{dt},j=1,2.$$

The quantity *λ_j_*(*t*)*dt *≈ *P *{*T *∈ *dt*, *X_T _*= *j*|*T *≥ *t *∪ (*T *≤ *t*, *X_T _*≠ *j*)} is then interpreted as the probability of experiencing a 0 → *j *transition at time *t*, among those who are either alive at time *t *or who have *experienced an event other than j at or prior to time t *[17]. As noted in [16] and pointed out in [17], this interpretation is problematic since it corresponds to a hazard for an improper random variable, as those individuals who experience an event other than *j *will have a 0 → *j *transition time of ∞. Further, it *conditions *on having reached one of the competing *terminal *states, which violates the intuitive notion that individuals who have reached a *terminal *state should not be considered for further follow-up (see [17], Principle 2). However, as the regression model developed by Fine and Gray in [16] provides a direct link between the cumulative incidence function and a set of covariates, in practice models based on the subdistribution hazard have proven useful [18,19].

### Estimators

For competing risks, the basic ingredients for estimation are the number of individuals who make a transition, and the number of individuals who are still *at risk *of making a transition, at each time point. Let *t*_1 _*< t*_2 _< ... <*t_k _*be the *k *distinct times where events occur in the data. Using standard counting process notation, let $N_{0j}\left(t\right)$ count the number of observed 0 → *j *transitions by time *t*, and *Y*_0_(*t*) indicate the number of individuals still in state 0 just prior to time *t*. The latter is called the number of individuals *at-risk *for transitions out of state 0 at time *t*, and naturally acounts for subjects that are right-censored by removing them from the risk set at their censoring time (it also accounts for *left-truncation*, where individuals do not enter the risk-set unless their event time occurs after a certain time). The total number of transitions by time *t *is given by *N*_0_. (*t*). Lastly, the number of 0 → *j *transitions at a given time *t *is given by $\Delta N_{0j}\left(t\right)$.

The starting point for estimation are the Nelson-Aalen (N-A) estimators of the cause-specific cumulative hazard functions (see [20], Chapter 4.2)

(4) $${\hat{A}}_{0j}\left(t\right)=\underset{i:t_{i}\leq t}{\sum }\frac{\Delta N_{0j}\left(t_{i}\right)}{Y_{0}\left(t_{i}\right)},j=1,2.$$

The N-A estimator of the all-cause hazard function is ${\hat{A}}_{0\cdot }\left(t\right)={\hat{A}}_{01}\left(t\right)+{\hat{A}}_{02}\left(t\right)$, and the increments of the N-A estimator are denoted $\Delta {\hat{A}}_{0j}\left(t\right)={\hat{A}}_{0j}\left(t\right)-{\hat{A}}_{0j}\left(t-\right)=\Delta N_{0j}\left(t\right)∕Y_{0}\left(t\right)$.

#### Kaplan-Meier Estimator

The standard Kaplan-Meier (KM) estimator [21] of the overall survivor function is then

(5) $$Ŝ\left(t\right)=\underset{i:t_{i}\leq t}{\prod }\left(1-\Delta {\hat{A}}_{0\cdot }\left(t_{i}\right)\right).$$

This estimator is a decreasing step function with "drops" at the observed transition times. The KM estimator estimates the probability that an event will eventually occur for *all *patients. For competing risks data, Ŝ(t) is then a valid estimate of the overall probability that an individual has not experienced *any *of the competing events by a given time.

By substituting ${\hat{A}}_{0j}\left(t\right)$ for ${\hat{A}}_{0\cdot }\left(t\right)$ in (5), an estimate of the *net *survival function $S_{j}\left(t\right)=\exp\left\{-A_{0j}\left(t\right)\right\}$ for event type *j *is obtained (see [20], Chapter 2.7). The complement $1-{Ŝ}_{j}\left(t\right)$ estimates the net probability to experience event *j *by a particular time. However, this net probability is only valid in the hypothetical setting where the competing events do not occur (reference [20], page 52). In the schematic represented by Figure 1, the quantity $1-{Ŝ}_{1}\left(t\right)$ estimates the probability of hospital discharge by time *t*, in an ideal world where in-hospital mortality cannot occur. In this case, individuals who experience one of the competing events are censored at their event times. The KM estimate for the probability of clinical stability and hospital discharge by time *t *will be denoted as $1-{Ŝ}_{cs}\left(t\right)$ and $1-{Ŝ}_{dis}\left(t\right)$, respectively. The variability of the KM estimator is given by the *Greenwood *estimator, which for the overall survival function has the form

(6) $$\hat{Var}\left\{Ŝ\left(t\right)\right\}={Ŝ}^{2}\left(t\right)\underset{i:t_{i}\leq t}{\sum }\frac{\Delta N_{0\cdot }\left(t_{i}\right)}{Y_{0}\left(t_{i}\right)\left\{Y_{0}\left(t_{i}\right)-\Delta N_{0\cdot }\left(t_{i}\right)\right\}},$$

(see [20], Chapter 4.2).

#### Cumulative Incidence Estimator

The Aalen-Johansen estimates of the cumulative incidence functions [22] are obtained by plugging in Ŝ(u-) for *P*(*T > u*-) and $\Delta {\hat{A}}_{0j}\left(u\right)$ for $\alpha_{0j}\left(u\right)du$ in (2), and summing over all observed transition times *t_i _*≤ *t*,

(7) $${\hat{CI}}_{j}\left(t\right)=\underset{i:t_{i}\leq t}{\sum }Ŝ\left(t_{i-1}\right)\Delta {\hat{A}}_{0j}\left(t_{i}\right),j=1,2.$$

This estimator can be derived in a straightforward fashion starting from the KM estimator of the overall survivor function [13]. The increments of the KM estimator (5) are $\hat{P}\left(T=t_{i}\right)=Ŝ\left(t_{i-1}\right)-Ŝ\left(t_{i}\right)=Ŝ\left(t_{i-1}\right)\Delta {\hat{A}}_{0j}\left(t_{i}\right)$. Since the cause-specific cumulative incidence functions sum to the all-cause distribution function, decomposing the KM increments into components corresponding to each hazard and summing over *t_i _*≤ *t*, gives the desired result. The estimate of the cumulative incidence function for clinical stability, hospital discharge, and in-hospital mortality by time *t *will be denoted as ${\hat{CI}}_{cs}\left(t\right)$, ${\hat{CI}}_{dis}\left(t\right)$, and ${\hat{CI}}_{mor}\left(t\right)$, respectively.

The variability of the cumulative incidence estimator has received considerable attention in the recent literature [13,23]. Braun and Yuan [23] compared six different estimators of the variance, and Allignol *et al*. [13] demonstrated that the Greenwood-type estimator of the variance (Equation (6) in [13]) is algebraically equivalent to the top performing estimators in [23]. Of note, the variance estimator given by Gray [15], available in the R package *cmprsk *[24], performed the poorest for small (*N *≤ 50) sample sizes.

#### Restricted Estimator

The first ad-hoc approach evaluated in this study was to exclude individuals who died from the analysis (restricted analysis). With this approach, individuals who die are excluded entirely from the analysis, and their LOS data are essentially treated as being censored at day zero. Of significant note is that the restricted estimator suffers from a serious conceptual flaw, in that estimation at any time *t *requires conditioning on the occurrence of *future *events after time *t *(see [17], Principle 1). Nevertheless, the complements of the KM estimates based on these data were used to estimate the probability of clinical stability and hospital discharge, denoted $1-{Ŝ}_{cs}^{R}\left(t\right)$ and $1-{Ŝ}_{dis}^{R}\left(t\right)$, respectively. The variance of this estimator is calculated using the Greenwood estimator on this restricted sample.

#### Worst Outcome Estimator

The second ad-hoc approach was to assign individuals who died the "worst" outcome (worst outcome analysis). In this approach, individuals who die are right-censored at the longest possible follow-up time. This is essentially equivalent to assigning a discharge or clinically stable time of ∞ to the individuals who die in the hospital, and so coincides with the random variable which forms the basis of the subdistribution hazard in Equation (3). The complement of the KM estimator based on these censored subdistribution times has been previously shown to be equivalent to the cumulative incidence estimator (see [25] and [26] for proofs, also noted in [15]). In the Appendix, we give a simplified algebraic proof of this equality for the special case of our situation, administratively right-censored data, and a heuristic argument is given in the 'CAPO Data' subsection of the Results. Estimates of the 'worst outcome' analysis for the probability of clinical stability and hospital discharge will be denoted $1-{Ŝ}_{cs}^{W}\left(t\right)$ and $1-{Ŝ}_{dis}^{W}\left(t\right)$, respectively. The estimate of the variability is again based on Greenwood's estimate.

### Confidence Intervals and Testing

Pointwise 95% confidence intervals for each estimator at time *t *can be based on the general formula $\text{Estimate(}t)\pm 1.96\sqrt{\hat{Var}\{\text{Estimate}(t)\}}$. For the KM, restricted, and worst outcome analysis, testing for differences in probability distributions between patient groups for LOS and TCS were done using the log-rank test [27], and denoted $\chi_{KM_{dis}}^{2}$, $\chi_{R_{dis}}^{2}$, and $\chi_{W_{dis}}^{2}$. For the competing risks approach, the test statistics presented in [15] were used for testing differences between cumulative incidence functions (denoted $\chi_{CI_{dis}}^{2}$). It should be noted that for the two ad-hoc approaches, the test statistics are used primarily for illustrative purposes, to demonstrate the potential differences in conclusions drawn based on the two approaches.

### CAPO Data

To illustrate the differences between the four estimation methods from an established database, analyses were performed on data from the international CAPO dataset (see [3,28] for details). We consider the subset of 1,635 patients aged 65 years or greater, who were admitted to the hospital with CAP between June 1, 2001 and January 1, 2007. The data set includes patients from 40 hospitals located in 10 countries on 4 continents, for a detailed listing of all participating hospitals see Additional File 1. CAP was defined as a new pulmonary infiltrate (within 24 hours of admission), and associated with at least one of the following factors: a new or increased cough, an abnormal temperature (*<*35.8°C or *>*37.8°C), or an abnormal leukocyte count (leukocytosis, leucopenia or the absence of immature neutrophils). Pneumonia was considered as community-acquired if a patient had no history of hospitalization during the two weeks prior to admission.

Time to clinical stability was defined using the American Thoracic Society criteria for switch therapy from intravenous to oral antibiotic therapy: 1) improvement in cough and shortness of breath; 2) afebrile status for ≥ 8 hours (*<*37.8°C); 3) normalizing leukocyte count by at least 10% from the previous day; and 4) adequate oral intake [29]. Time to clinical stability was calculated in days as the time from admission to the hospital to the time the above four criteria were met. Length of hospital stay (LOS) was defined as the time from admission to the hospital to discharge from the hospital. LOS was right censored at 30 days, since hospital stays longer than 30 days are considered unrelated to CAP and are not of primary interest [28]. Classification of patients into risk classes was done using the Pneumonia Severity Index (PSI) [30]. There are five possible rankings for the PSI, labeled Risk Class (RC) I to V, from least to most severe.

The study was approved by the Human Subject Protection Program Institutional Review Board at the University of Louisville. Additional approval was obtained from the local internal review board for each participating hospital. Patient consent was waived due to the retrospective and observational study design.

### Simulated Data

Competing risks data were simulated following the methodology outlined in Beyersmann *et al*. [31]. A discrete time scale was used for *t*, in units of days. Two non-parametric hazard functions were constructed that approximated the observed discharge distributions in the CAPO data for Risk Class V patients:

$$\begin{array}{c} \alpha_{01}\left(t\right)=P\left(T=t,X_{T}=1|T\geq t\right)=0.32∕\left(t+1\right)\\ \alpha_{02}\left(t\right)=P\left(T=t,X_{T}=2|T\geq t\right)=\left\{\begin{array}{cc} 0.06∕\left(t+1\right) & t<15\\ 0.0003+0.001t & t\geq 15 \end{array}\right) \end{array}$$

Conditional on an event happening at time *t*, a binomial probability

*P*(*X_T _*= *i*|*T *= *t*) = *α*_0*i*_(*t*)/{*α*_01_(*t*) + *α*_02_(*t*)} was used to determine the event type, discharge (*i *= 1) or death (*i *= 2).

Events were simulated for *t *= 1,..., 30, and all individuals who had not experienced an event by day 30 were right-censored at that point. For each simulation, we obtained four estimates of the probability of discharge by day *t*, including the cumulative incidence estimator ${\hat{CI}}_{dis}\left(t\right)$, the complement of the Kaplan-Meier estimator $1-{Ŝ}_{dis}\left(t\right)$, and the two ad-hoc estimators corresponding to the restricted analysis (all patients who died were removed, $1-{Ŝ}_{dis}^{R}\left(t\right)$) and the 'worst outcome' analysis (all patients who died were right-censored at the longest allowed length of stay of 30, $1-{Ŝ}_{dis}^{W}\left(t\right)$). A listing of all four estimators is given in Table 1. To evaluate point estimates for each of the etimators, a total of 1,000 Monte Carlo simulations were run with a patient sample size of 1,500 for each simulation. These estimates were compared with the true cumulative incidence function *CI_dis_*(*t*).

**Table 1:** Description of methods evaluated using simulated data

Estimator	Description
$1-{Ŝ}_{dis}\left(t\right)$	Complement of the Kaplan-Meier estimator of hospital discharge, obtained by censoring those patients who died at their time of death

${\hat{CI}}_{dis}\left(t\right)$	Estimate of cumulative incidence function of hospital discharge, obtained by treating in-hospital mortality as a competing risk

$1-{Ŝ}_{dis}^{R}\left(t\right)$	Complement of the KM estimator of hospital discharge, obtained by removing those patients who died

$1-{Ŝ}_{dis}^{W}\left(t\right)$	Complement of the KM estimator of hospital discharge, obtained by censoring those patients who died at the longest recorded LOS (30 days)

We additionally compared the coverage of 95% linear confidence intervals for *CI_dis_*(*t*) based on ${\hat{CI}}_{dis}\left(t\right)\pm 1.96\sqrt{\hat{Var}\left\{{\hat{CI}}_{dis}\left(t\right)\right\}}$, using both Gray's estimate [15] and the Greenwood-type estimate of the variance (Equation (6) of [13]). An additional 1,000 simulations were generated with sample sizes of 25, 50, 100, 200, and 750 to compare the coverage rates of the two estimators.

The probability of rejecting the null hypothesis of no differences in hospital discharge probability distributions between two patient groups was evaluated under several scenarios. First, simulations were conducted under the null hypothesis, with both the mortality and discharge hazard functions being equal between the two patient groups. Additionally, we ran simulations with the hazard ratio for mortality (HR*_mor_*) equal to 1.15 and 1.5, to investigate the dependence of each test statistic on the hazard ratio of the competing event. Five different sets of simulations were conducted under the alternative hypothesis, that discharge hazard functions differed between the two patient populations. In each case, a hazard ratio for hospital discharge HR*_dis _*of 1.15 was used. The five scenarios were a) the mortality hazard rates were equal for the two groups, b) the patient group with a higher discharge rate also had a slightly lower mortality rate (HR*_mor _*= 1/1.15 = 0.87), c) the patient group with a higher discharge rate also had a significantly lower mortality rate (HR*_mor _*= 1/1.5 = 0.67), d) the patient group with a higher discharge rate also had a slightly *higher *mortality rate (hazard ratio for mortality HR*_mor _*= 1.15), and e) the patient group with a higher discharge rate also had a significantly higher mortality rate (HR*_mor _*= 1.5). The latter two situations, though somewhat counterintuitive, may correspond to a therapy which reduces LOS but has a deleterious side effect in some patients which increases mortality. One thousand Monte Carlo replicates were used in each case, with a sample size of 750 patients for each group. An *α *= 0.05 cutoff was used for each test.

### Software

All the analyses presented in this paper were performed using R version 2.12.2 [32], which is a freely available, open-source statistical programming language. The functions for competing risks analysis are included in the R add-on package *cmprsk *[24], which also includes functions for fitting proportional-hazards regression models for the subdistribution function of a competing risk [16]. A nice tutorial on using the *cmprsk *package is given in [33]. Limitations of the *cmprsk *package are that it can only handle right-censored data and that it uses an estimate of the variance which has been shown to be suboptimal [23]. Alternative R packages which can handle left-truncated data and use the Greenwood-type estimate of the variance include *etm *[34], *msSurv *[35], and *mstate *[36]. These are specialized packages for multi-state models, of which competing risks is a special case. However, only the *cmprsk *package includes test statsitics [15] for comparing cumulative incidence curves. The interested reader is directed to the CRAN task view on survival [37] for a detailed listing of available R-packages for survival analysis. Additional Files 2, 3, and 4 for this manuscript contain documented R code and data illustrating how to carry out all the competing risks analysis performed in this paper.

## Results

### An Illustrative Example

To illustrate the differences between the five estimators, consider the following artificial example of ten patients who are admitted to the hospital with CAP. Suppose that two of the patients die at days 3 and 5, five of the patients reach clinical stability on days 2, 2, 4, 5, and 7, and three patients do not reach clinical stability by day 7. After day 7, information regarding clinical stability is no longer collected, so that the three patients who did not reach clinical stability by day 7 are censored at day 7. The data are displayed in Tables 2, 3, where each row indicates a unique event time. Here *t_i _*indicates the event time in days (either death or reaching clinical stability), *s_i _*indicates the number of patients who reached clinical stability at time *t_i_*, *d_i _*indicates the number of patients who died at time *t_i_*, and *y_i _*indicates the number of patients who are eligible to either die or reach clinical stability at time *t_i_*. Table 2 gives the estimates based on the complement of the Kaplan-Meier estimator and the cumulative incidence function, while Table 3 provides estimates based on the ad-hoc estimators. The complement of the KM estimator $1-{Ŝ}_{cs}\left(t\right)$ is uniformly higher than the cumulative incidence estimator ${\hat{CI}}_{cs}\left(t\right)$, and the clinical interpretation of this estimate is unclear since it corresponds to an "alternative" world where patients cannot die in the hospital (see [17], Principle 3). Since the complement of the KM estimator assumes that all patients who are censored are still eligible to experience the event at some point in the future, it will overestimate the probability of experiencing the event of interest (reaching clinical stability) when competing risks are present (mortality).

**Table 2:** Kaplan-Meier and cumulative incidence estimators of probability of clinical stability for artificial data

				**Kaplan-Meier**			**Cumulative Incidence**		
*t_i_*	*y_i_*	*s_i_*	*d_i_*	${\hat{S}}_{cs}\left(t_{i}\right)$	$1-{\hat{S}}_{cs}\left(t_{i}\right)$	SE	$\hat{S}\left(t_{i}\right)$	${\hat{CI}}_{cs}\left(t_{i}\right)$	SE
2	10	2	0	1-2/10 = 0.80	0.20	0.126	1-2/10 = 0.8	2/10 = 0.2	0.133
3	8	0	1	0.80	0.20	0.126	0.8*(1-1/8) = 0.7	0.2+0/8*(0.8) = 0.2	0.133
4	7	1	0	0.8*(1-1/7) = 0.69	0.31	0.151	0.7*(1-1/7) = 0.6	0.2+1/7*(0.7) = 0.3	0.154
5	6	1	1	0.69*(1-1/6) = 0.57	0.43	0.164	0.6*(1-2/6) = 0.4	0.3+1/6*(0.6) = 0.4	0.167
7	4	1	0	0.57*(1-1/4) = 0.43	0.57	0.174	0.4*(1-1/4) = 0.3	0.4+0/4*(0.4) = 0.5	0.172
>7	3			0.43	0.57		0.3	0.5	

*t_i _*= *i*th time point; *y_i _*= number at risk at time *t_i_*; *s_i _*= number clinically stable at time *t_i_*; *d_i _*= number who died at time *t_i_*; $Ŝ\left(t_{i}\right)=\text{event}\;\text{free}\;\text{survival}\;\text{at}\;\text{time}\;t_{i}$ = event free survival at time *t_i_*; SE = standard error

**Table 3:** Ad-hoc estimators of probability of clinical stability for artificial data

				**Restricted Analysis**			**Assign Worst Outcome**		
*t_i_*	*y_i_*	*s_i_*	*d_i_*	${\hat{S}}_{cs}^{R}\left(t_{i}\right)$	$1-{\hat{S}}_{cs}^{R}\left(t_{i}\right)$	SE	${\hat{S}}_{cs}^{W}\left(t_{i}\right)$	$1-{\hat{S}}_{cs}^{W}\left(t_{i}\right)$	SE
2	10	2	0	1-2/8 = 0.75	0.25	0.153	1-2/10 = 0.8	0.2	0.126
3	8	0	1	0.75	0.25	0.153	0.8	0.2	0.126
4	7	1	0	0.75*(1-1/6) = 0.63	0.37	0.171	0.8*(1-1/8) = 0.7	0.3	0.145
5	6	1	1	0.63*(1-1/5) = 0.50	0.5	0.177	0.7*(1-1/7) = 0.6	0.4	0.155
7	4	1	0	0.5*(1-1/4) = 0.38	0.62	0.171	0.6*(1-1/6) = 0.5	0.5	0.158
> 7	3			0.38	0.62		0.5	0.5	

*t_i _*= ith time point; *y_i _*= number at risk at time *t_i_*; *s_i _*= number clinically stable at time *t_i_*; *d_i _*= number who died at time *t_i_*; SE = standard error

The estimate of clinical stability restricted to only patients who lived, $1-{Ŝ}_{cs}^{R}\left(t\right)$, is higher than any of the other estimates. Since this estimate conditions on patients who die in the hospital *at some future time*, it is difficult to imagine how such an estimator would be useful in practice. The admitting physician would need to "know" which patients would ultimately die and which would live *at the time of admission*. In the strictly hypothetical situation that this knoweldge could be possessed, it would seem equally likely that the physician would know the exact day that clinical stability would be reached, precluding the need for this type of analysis.

The 'worst outcome' estimator $1-{Ŝ}_{cs}^{W}\left(t\right)$ coincides with the cumulative incidence estimator ${\hat{CI}}_{cs}\left(t\right)$. A heuristic for understanding the equivalence between the two algorithms involves Efron's 'redistribution to the right' algorithm [38]. Efron demonstrated that the Kaplan-Meier estimator can be derived using an algorithm which uniformly redistributes the probability mass associated with each right censored observation to all times to its right. When patients who die are not censored at the point of death but are instead censored at the end of the follow-up time (7 days), their probability weight is not re-distributed to the other patients who are still alive and have not been discharged. Hence, the jump sizes for the cumulative incidence estimator and 'worst outcome' estimator are equivalent, whereas the jump sizes of the complement of the KM estimator are augmented by the redistributed weight of past censored observations.

The standard error of the cumulative incidence function is Gray's estimate [15] reported by the *cmprsk *package, and is notably larger than the standard error for the 'worst outcome' estimator. The latter is based on Greenwood's estimate, which we empirically verified is equivalent to the Greenwood-type estimate of the variance for competing risks (Equation (6) in [13]). Differences between these two estimates of the variance, in terms of coverage rates for confidence intervals of point estimates, are investigated further in the Simulated Data section.

### CAPO Data

**Figure 2:**
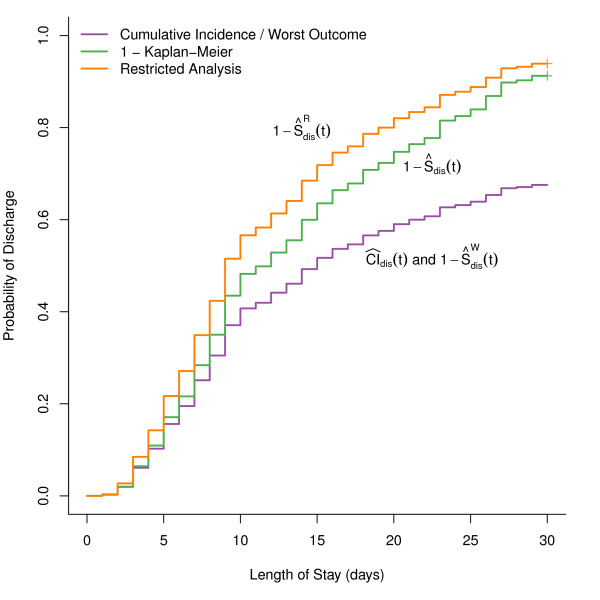
**Estimates for probability of hospital discharge**. Four different estimators for the probability of hospital discharge, for elderly patients hospitalized with CAP with PSI Risk Class V. The four approaches outlined in the text were used: cumulative incidence estimator and complement of the Kaplan-Meier estimator when patients who died were censored at the longest LOS value of 30 days (${\hat{CI}}_{dis}\left(t\right)$ and $1-{Ŝ}_{dis}^{W}\left(t\right)$, purple line), complement of the Kaplan-Meier estimator ($1-{Ŝ}_{dis}\left(t\right)$, green line), and complement of the Kaplan-Meier estimator restricted to only patients who survived ($1-{Ŝ}_{dis}^{R}\left(t\right)$, orange line).

Our analysis of the CAPO data focuses on length of hospital stay. We initially restrict our presentation to the subset of patients in the highest risk class calculated from the pneumonia severity index (PSI), Risk Class V (*n *= 410 patients). Figure 2 presents the four estimates for the probability of being discharged by a given day after hospital admittance. Since the in-hospital mortality is relatively high in this patient subset (20% by day 14), the complement of the Kaplan-Meier estimate is considerably higher than the cumulative incidence estimate. The estimator restricted to the subset of patients who survived (restricted analysis) gives an overly-optimistic picture of the probability of being discharged, and as noted previously the practical use of this estimator is highly questionable. The 'worst outcome' estimator again coincides with the cumulative incidence estimator.

The test-statistic for hospital discharge based on the cumulative incidence function has a p-value of 5 × 10^-17^. Log-rank tests based on the ad-hoc estimators, restricted and worst outcome, give p-values of 8 × 10^-4 ^and 3 × 10^-18 ^respectively, which are several orders of magnitude different. The log-rank test based on the Kaplan-Meier estimator gives a p-value = 8 × 10^-8^, but it is unclear what is the clinical interpretation of this test.

**Figure 3:**
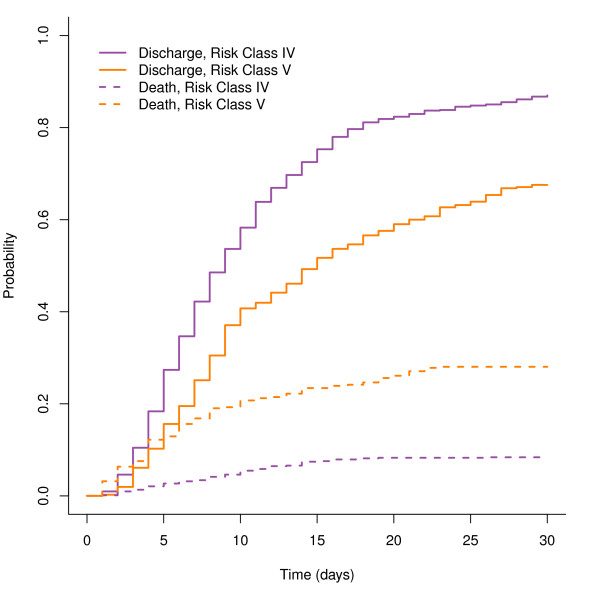
**Cumulative incidence curves for discharge and in-hospital mortality**. Estimated cumulative incidence curves for hospital discharge and in-hospital mortality, for elderly patients hospitalized with CAP with PSI Risk Class of IV or V.

The competing risks analysis allows simultaneous comparison of both LOS and mortality incidence. In contrast, right censoring or removing patients who die prevents mortality information from being incorporated. Treating mortality as a competing risk provides a mechanism to view the incidence curves of both outcomes, so that multiple outcomes can be compared between patient groups. To illustrate, we view both the discharge and mortality incidence for patients in RC V (410 patients) versus RC IV (822 patients) in Figure 3. The patients in RC IV have the higher discharge incidence curve, indicating that there is a higher probability of being discharged on any given day relative to patients in RC V. Conversely, patients in RC V have the higher in-hospital mortality incidence curve.

### Simulated Data

**Figure 4:**
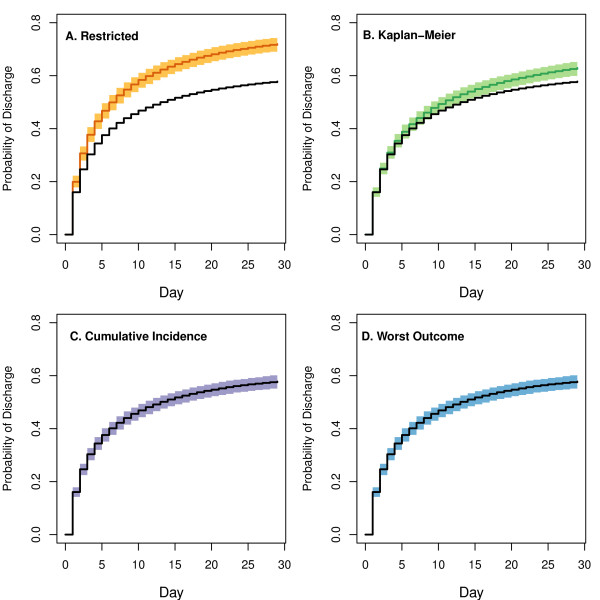
**Probability of hospital discharge from simulated data**. Median values of each estimation method for the probability of hospital discharge, based on 1,000 simulations, as detailed in the 'Simulated Data' section of the Methods. The underlying cumulative incidence curves are shown in solid black lines. Median estimates of the restricted analysis estimator $1-{Ŝ}_{dis}^{R}\left(t\right)$ (top left panel, orange curve) and the complement of the Kaplan-Meier estimator $1-{Ŝ}_{dis}^{W}\left(t\right)$ (top right panel, green curve) each overestimate the probability of discharge. Median estimates from the cumulative incidence estimator ${\hat{CI}}_{dis}\left(t\right)$ (purple, lower left panel) and the worst outcome estimator $1-{Ŝ}_{dis}^{W}\left(t\right)$ (blue, lower right panel) coincided exactly with the true cumulative incidence function, as was expected. Shaded polygons give the 97.5% and 2.5% percentiles of the estimates from the simulations, for each time point.

To more comprehensively evaluate the differences between the four methods, we simulated competing risks data using the methods in Beyersmann *et al*. [31]. The hazard functions for discharge and in-hospital mortality were selected to roughly match the observed hazards in our data for Risk Class V patients (see Methods). Figure 4 presents the median values and 2.5% and 97.5% percentiles for each point estimate based on the 1,000 simulations for each estimation method, using a sample size of 1,500 for each simulation. The true cumulative incidence curve is shown in a solid black line for each plot. As expected, the median of the cumulative incidence estimates and the worst outcome estimates coincide exactly with the underlying cumulative incidence function. The median estimates for the restricted estimator and complement of the Kaplan-Meier estimator are also shown, and display a similar pattern to that observed in the real data.

As noted in the Methods, their are several estimators of the variance of cumulative incidence estimator that have been proposed in the literature. Previous research [23] has demonstrated that the Greenwood-type estimator has better performance for small sample sizes, in particular relative to the estimator proposed by Gray [15]. We evaluated the coverage rates of 95% confidence intervals for the true cumulative incidence at selected time points, for both the Greenwood estimator of the variance and the Gray estimator. Results are displayed in Table 4 and show that the coverage rates for both intervals are in very good agreement with each other and close to the nominal 95% level for the range of sample sizes evaluated. Thus, though Gray's estimate of the variance is higher for smaller sample sizes, this difference did not translate into a difference in coverage rates for our simulations.

**Table 4:** Coverage probabilities for 95% confidence intervals for *CI*(*t*), based on the cumulative incidence estimator (${\hat{CI}}_{dis}\left(t\right)$) using either Gray's estimate or the Greenwood-type estimate of the variance

		**Time (days)**				
Sample Size	Estimator	5	10	15	20	25
1500	Gray	0.945	0.952	0.951	0.956	0.954
	Greenwood	0.945	0.954	0.951	0.957	0.954
750	Gray	0.953	0.958	0.962	0.965	0.961
	Greenwood	0.960	0.963	0.962	0.965	0.961
200	Gray	0.935	0.938	0.943	0.942	0.944
	Greenwood	0.935	0.938	0.953	0.942	0.944
100	Gray	0.961	0.949	0.952	0.955	0.950
	Greenwood	0.961	0.949	0.949	0.955	0.943
50	Gray	0.938	0.950	0.926	0.931	0.938
	Greenwood	0.938	0.950	0.926	0.931	0.938
25	Gray	0.950	0.941	0.936	0.955	0.936
	Greenwood	0.950	0.941	0.936	0.955	0.936

Lastly, we evaluated the power for the four methods to reject the null hypothesis of no differences in discharge probability distributions between patient groups, under various scenarios. A sample size of 750 patients was used for each of the two patient groups, for each simulation. It should be noted that each method is testing a different null hypothesis, e.g. the test based on the cumulative incidence estimator is testing for differences between the cumulative incidence functions *CI_j_*(*t*), *j *= 1, 2, while the test based on the Kaplan-Meier estimator is testing for differences between *S_j_*(*t*), *j *= 1, 2. Hence the results based on the tests are not *directly *comparable with each other. However, the simulations are still useful for illustrating how tests based on the estimators perform under different situations.

The first row of Table 5 displays the rejection proportions for the four methods when the hazards of both mortality and discharge are equal between the two patient groups (i.e., the null hypothesis is true), using an *α *level of 0.05. The methods all have a rejection rate close to the nominal level of 0.05. The next two rows display the rejection proportions when the hazard of discharge is the same, but the mortality hazard differs between the two groups. As the hazard of the competing event will have an influence on the incidence of the event of interest, all of the test statistics (except the KM-based test statistic $\chi_{KM_{dis}}^{2}$) increase as the HR for mortality deviates further from one. The KM-based test statistic is not influenced by the HR for mortality since it treats mortality events as independent right-censoring events (*i.e*., it assumes mortality and discharge are independent).

**Table 5:** Proportion of times that the null hypothesis of no difference in discharge probabilities is rejected, using an *α *= 0.05 significance level

		**Test**			
HR*_dis_*	HR*_mor_*	$\chi_{CI_{dis}}^{2}$	$\chi_{W_{dis}}^{2}$	$\chi_{KM_{dis}}^{2}$	$\chi_{R_{dis}}^{2}$
1.0	1.0	0.048	0.047	0.049	0.054
	1.15	0.064	0.061	0.055	0.062
	1.5	0.128	0.120	0.048	0.362

1.15	1.0	0.553	0.544	0.583	0.505
	0.67	0.772	0.772	0.601	0.137
	0.87	0.652	0.644	0.603	0.339
	1.15	0.470	0.462	0.577	0.681
	1.5	0.256	0.248	0.577	0.946

HR*_dis _*= hazard ratio of discharge; HR*_mor _*= hazard ratio of mortality

The next five rows of Table 5 display the rejection proportions when the hazard of discharge is different between the two groups, for varying hazard ratios of mortality. When the hazard of mortality is the same between the two patient populations, the power of the methods to reject the null hypothesis of differences in discharge probability distributions are fairly close to each other. When the hazard ratio for mortality is varied, an interesting pattern emerges. When the hazard ratio is below one, the patient group with the higher discharge rate also has a decreased mortality rate, leading to more patients being discharged in that group and fewer patients dying. Hence, the difference in cumulative incidence curves for discharge is *increased *between the two patient groups, and the power subsequently increases as well. Conversely, when the hazard ratio for mortality is above one, the patient group with the higher discharge rate also has an *increased *mortality rate. Thus, patients in this group have an overall higher rate of *either *event, and since more patients are dying in this group as well the difference in the *cumulative incidence *of discharge is decreased relative to the second group. This is particularly true when the HR*_mor _>*HR*_dis_*, since in this case the *ratio *of discharge hazard (*α*_01_) to mortality hazard (*α*_02_) for this group is *lower *relative to the ratio of these two hazards for the second group.

The trend for $\chi_{R_{dis}}^{2}$, the test based on the restricted analysis estimator, is the exact opposite. When the HR for mortality is above one, the group with the higher discharge rate will have more patients *removed *from the study due to mortality. Hence the *cumulative incidence *of discharge calculated on this restricted sample will be exaggerated, leading to a greater difference in discharge cumulative incidence between the two patient groups. Conversely, when the HR for mortality is below one, *fewer *patients will be removed from the study for the group with the higher discharge rate, and the cumulative incidence of discharge will be *dampened *on this restricted sample, leading to a decrease in differences in discharge cumulative incidences between the two groups. The log-rank test based on the KM estimator is again unaffected by the changes in mortality hazard between the two patient groups.

## Discussion

The way in which mortality is handled while investigating other time-related outcomes can have an impact on the results obtained and the interpretations conjectured. In the CAP literature, there is no consensus for how to properly handle mortality when evaluating TCS and LOS. Therefore, direct comparisons are difficult and conflicting conclusions can be reached depending on the approach that is used. Two ad-hoc approaches for handling mortality were investigated in this paper, analysis restricted to the subset of patients who survived and assigning patients who died the worst possible outcome. The two methods give different results for the same data and could lead to conflicting conclusions, unless investigators are aware of the differences between the estimators. The first approach conditions on the occurrence of future events, and the practical use of this estimator is questionable. The second approach coincides with the Kaplan-Meier estimator based on the subdistribution hazard, which has been proven to be equivalent to the cumulative incidence estimator [25]. In both cases the information concerning time to in-hospital mortality is ignored, preventing dual-assessment of both time to clinical stability or discharge and patient mortality.

It should be mentioned that although we considered the 'worst outcome' approach to be ad-hoc, the equivalence of this method to the random variables defined for the subdistribution function in [15] does not mean that we consider that latter to be ad-hoc as well. Indeed, the characterization of the subdistribution hazard function by Gray had a clearly defined purpose and context. In addition to showing equivalence between the KM estimator based on the subdistribution hazard and the cumulative incidence estimator, Geskus [25] showed that the latter could be characterized as a weighted empirical cumulative distribution function. In both cases, the weights depend on the censoring and truncation distributions of the data. A third characterization of the cumulative incidence function is as a KM estimator with a 'fractional risk set' [39], where censored individuals contribute a fractional mass to the risk set specified by an estimate of the probability that the individual would have experienced a particular event type. Applications of these alternative characterizations include regression modeling based on the subdistribution hazard [25], and non-parametric estimation for multi-state models [40,41].

The complement of the Kaplan-Meier estimator, which censors patients who die at their time of death, provides an estimate that is between the cumulative incidence function and restricted analysis estimator. As past authors have pointed out [17,20,42], this curve lacks a reasonable clinical interpretation in the presence of competing risks, since patients who die are still considered eligible to reach clinical stability and be discharged from the hospital. Proponents of the KM estimator favor the fact that it focuses on a single event type, and argue that the cumulative incidence function is difficult to interpret on its own due to its dependence upon the incidence of the competing events [43]. In response to this, Pepe and Mori [42] proposed the *conditional probability estimator*, which estimates the probability of an event conditional on the other events having not occurred by a given time. However, this estimator has been criticized on a conceptual basis [17], in that conditioning on the non-occurrence of the other event types either conditions on the future (when considered from the origin time) or on having reached / not reached a terminal event (when considered at the current time *t*). As a result, we did not consider the conditional probability estimator in this manuscript.

In contrast to the ad-hoc estimators and Kaplan-Meier estimator, the estimators based on treating in-hospital mortality as a competing risk have clearly defined interpretations. Recent applications of these approaches (and more general multi-state models) to the study of nosocomial (hospital-acquired) pneumonia infections have been conducted by Beyersmann *et al*. [1,12,44] and Wolkewitz *et al*. [45]. However, there is still clear evidence that these methods are not commonly used in situations where they are warranted. Competing risks models are intuitively attractive because they disallow patients who die to subsequently be considered eligible for discharge or clinical stability (see [17], Principle 2). Further, the estimator also estimates the probability of in-hospital death, so that an investigator can simultaneously evaluate the probability of reaching clinical stability or hospital discharge versus death by any given time.

Simulations revealed that the power of each method to detect differences in underlying discharge rates between patient groups depended on the rate of the competing event (mortality). When the hazard ratios were in opposite directions, so that the patient group with the higher discharge rate also had a lower mortality rate, differences in the cumulative incidence of discharge were increased and the power based on Gray's test [15] for the differences in cumulative incidence curves also increased. However, when the patient group with the higher discharge rate also had a *higher *mortality rate, the difference between cumulative incidence curves for discharge decreased with a corresponding decrease in power based on Gray's test. This phenomenon was explored in greater detail in Allignol *et al*. [10], who stressed the importance of the baseline hazard rate of each event type in addition to the hazard ratios. This importance was exemplified by Beyersmann *et al*. ([12], pgs. 336-337), who demonstrated that conflicting results between the incidence rate and the incidence proportion (cumulative incidence) can occur when the hazard rate for the competing event dominates the hazard for the event of interest, and the differences in hazards for the event of interest are minor in magnitude. In our case, if we consider mortality to be the event of interest, such a case could arise if the hazard for mortality was minor in magnitude relative to the hazard for discharge / clinical stability. Then, if a subset of patients had a reduced mortality rate but also a reduced discharge rate, then patients in this group will on average have longer times in the hospital and, eventually, the *cumulative incidence *of mortality will be higher for these patients. Such a situation can be readily resolved by plotting the Nelson-Aalen estimators of the cumulative hazards, *c.f*. Figure 2 in [12] and [10]. Fortunately, in most real-world situations the patient group with the higher discharge rate will also have a *reduced *mortality rate, so that interpretation of results in most cases will be easier.

In contrast to the tests based on the cumulative incidence function, tests based on restricting the patient sample to those who survived had increased "power" when the group with the higher discharge rate also had a higher mortality rate. However, by removing patients who died from the study, the differences in mortality rates between the two patient populations results in a *biased *comparison between the two patient groups. The motivation for restricting analysis to the subset of patients who survived results from the desire of investigators to compare outcomes (LOS and TCS) seperately among this patient population. But when mortality differs between patient groups (e.g., as determined by treatment assignment), comparisons restricted to patients who survived will not result in a *compatible *set of patients being compared between the two groups. An intriguing alternative to the methods evaluated in this article which addresses this issue is the "survivor average causal effect" (SACE) [46,47]. This method was designed to estimate a treatment effect on an outcome variable (e.g., LOS), when some of the patients die during the course of evaluation. Mortality is handled via *stratification*, but not on the *observed *outcome, which can be affected by treatment assignment. Rather, stratification is on the *dual outcome *of survival and treatment assignment. The primary focus is to estimate the treatment effect for the *principal stratum*, that is, those who would have survived irrespective of treatment assignment. Since for each individual survival is only observed for the actual treatment assigned, estimation of 'potential outcomes', or outcomes for each patient if they were given the opposite of their assigned treatment, is needed [48,49]. Comparisons between SACE and the competing risks methods discussed in this article would be an interesting area of future research.

In our modeling of TCS, we focused solely on the time from hospital admission to clinical stability and included in-hospital mortality as a competing risk. Hospital discharge was not included as an additional competing risk, which is reasonable given the definition of time of clinical stability as the time the criteria for switching from intravenous to oral antibiotic therapy was met. In the competing risks model, each outcome is treated as an absorbing state, so that transitions do not occur after reaching these outcomes. In interest is solely in the time that clinical stability is reached, then this model is reasonable. However, an extended modeling for clinical stability and subsequent discharge and/or in-hospital mortality can be constructed, using a multi-state model which allows transitions from the clinically stable state to these other two outcomes. The model would be similar to that used for modeling nosocomial infections in Beyersmann *et al*. [12], *c.f*. Figure 1 in that reference. In such a model, the incidence rate, the incidence proportion, and the *prevalence *of patients who are clinically stable at/by a given time can all be addressed. However, when competing risks are present, care must be taken in intrepreting the results, as the rate of the competing event (mortality) can result in apparently conflicting results between the incidence rate and proportion of clinically stable patients (see [12], Section 3.2, for a detailed discussion and analysis).

Lastly, in our evaluation of methods for handling mortality we did not discuss the issue of incorporating additional risk factors, which can be done using a regression model. When competing risks are present, regression modeling focuses on either Cox models [50] for the cause-specific hazards, or modeling the effect of covariates on the cumulative incidence function [16,51]. However, modeling based on cause-specific hazards are difficult to interpret in terms of the effect on the cumulative incidence function, as all cause-specific hazards models must be considered simultaneously. In contrast, proportional cause-specific hazards does not imply proportional subdistribution hazards, and when the former holds models based on the latter criterion will be mis-specified [52]. Extensive comparisons between the two methods have been conducted by several authors [52,53], and Grambauer *et al*. [52] offers a robust approach for interpreting subdistribution models based on a time-averaged effect of the subdistribution hazard ratio (the *least false parameter*).

## Conclusions

This paper investigates mechanisms for handling in-hospital mortality when analyzing length of hospital stay (LOS) or time to clinical stability (TCS), using data from patients admitted to the hospital with community-acquired pneumonia. Two currently used ad-hoc approaches, restricting analysis to those patients who lived and assigning individuals who die the worst outcome (longest LOS or TCS), gave disparate results when applied to the same data set and are discouraged from use. In contrast, estimators based on treating mortality as a competing risk have clinically relevant interpretations. Additionally, the incidence of mortality can be compared simultaneously with that of discharge / clinical stability, for more comprehensive comparisons between patient populations. These estimators have been readily available in the literature for over thirty years, yet still are frequently overlooked when analyzing time-to-event outcomes in the presence of competing risks. With the ready availability of software for these estimators and their straightforward interpretation, there is no reason to eschew them in favor of other ad-hoc estimators that may be considered. We provide illustrative statistical code as supplemental material for investigators to use in their own studies, to promote use of these estimators in practice and improve compatibility between studies that are investigating these time-to-event outcomes. Interested readers are also referred to the many excellent articles in the literature [1,10,12,14] for follow-up on this topic.

## Abbreviations

The following is a list of the abbreviations used throughout the manuscript: **CAP: **Community-Acquired Pneumonia; **CAPO: **Community-Acquired Pneumonia Organization; **CI: **Cumulative Incidence; **CRAN: **Comprehensive R Archive Network; **HR: **Hazard Ratio; **KM: **Kaplan-Meier; **LOS: **Length of Stay; **PSI: **Pneumonia Severity Index; **RC: **Risk Class; and **TCS: **Time to Clinical Stability.

## Competing interests

The authors declare that they have no competing interests.

## Authors' contributions

GNB wrote the manuscript, performed the data analysis, and created the supplemental material. CB performed the simulation studies. JAR provided the CAPO data and clinical insight to the problem. JM originally conceived of the problem and supervised the research. All authors helped with drafting the manuscript, and read and approved the final draft.

## Appendix

### Equivalence of 'worst outcome' and cumulative incidence estimators

The complement of the KM estimator based on the subdistribution hazard has been previously shown to be equivalent to the cumulative incidence estimator [25,26], accounting for both general right-censoring and left-truncation. Here, we give a simplified proof of this equality for the special case of administratively right-censored data (that is, the data are fully observed until the end of the follow-up period). It is evident that the two estimators ${\hat{CI}}_{1}\left(t\right)$ and $1-{Ŝ}_{1}^{W}\left(t\right)$ have jumps at the same event times, therefore it is sufficient to prove equality of the jump sizes to prove equality of the two estimators. That is, we need to show that

(8) $$Ŝ\left(t_{i-1}\right)\frac{\Delta N_{01}\left(t_{i}\right)}{Y_{0}\left(t_{i}\right)}={Ŝ}_{1}^{W}\left(t_{i-1}\right)\frac{\Delta N_{01}\left(t_{i}\right)}{Y_{0}^{*}\left(t_{i}\right)}$$

for all times *i *= 1,..., *k*, where $Y_{0}^{*}\left(t_{i}\right)=Y_{0}\left(t_{i}\right)+N_{02}\left(t_{i-1}\right)$.

The proof goes by induction. Consider the first jump (i.e., the first 0 → 1 transition), at time $t_{i^{*}}$. If the first overall event is a 0 → 1 transition, then the two jump sizes are trivially equal since $Ŝ\left(t_{i^{*}}-\right)={Ŝ}_{1}^{W}\left(t_{i^{*}}-\right)=1$ and $Y_{0}\left(t_{i}\right)=Y_{0}^{*}\left(t_{i}\right)=N$, the initial sample size. If instead the first observed transition is 0 → 2, then the right-hand side (RHS) of (8) is $\left\{Y_{0}\left(t_{i^{*}}\right)∕N\right\}\left\{\Delta N_{01}\left(t_{i^{*}}\right)∕Y_{0}\left(t_{i^{*}}\right)\right\}=\Delta N_{01}\left(t_{i^{*}}\right)∕N$, equivalent to the LHS. Assuming equality of the jumps holds for time *t*_*i*_, then for time *t*_*i*+1 _we have for the RHS of (8):

$$\begin{array}{llll} Ŝ\left(t_{i}\right)\frac{\Delta N_{01}\left(t_{i+1}\right)}{Y_{0}\left(t_{i+1}\right)} & =Ŝ\left(t_{i-1}\right)\left(1-\frac{\Delta N_{0\cdot }\left(t_{i}\right)}{Y_{0}\left(t_{i}\right)}\right)\frac{\Delta N_{01}\left(t_{i+1}\right)}{Y_{0}\left(t_{i+1}\right)}\; & & \;\\ & =\frac{{Ŝ}_{1}^{W}\left(t_{i-1}\right)Y_{0}\left(t_{i}\right)}{Y_{0}^{*}\left(t_{i}\right)}\left(1-\frac{\Delta N_{0\cdot }\left(t_{i}\right)}{Y_{0}\left(t_{i}\right)}\right)\frac{\Delta N_{01}\left(t_{i+1}\right)}{Y_{0}\left(t_{i+1}\right)}\; & & \;\\ & =\frac{{Ŝ}_{1}^{W}\left(t_{i-1}\right)\Delta N_{01}\left(t_{i+1}\right)}{Y_{0}^{*}\left(t_{i}\right)Y_{0}\left(t_{i+1}\right)}\left(Y_{0}\left(t_{i}\right)-\Delta N_{0\cdot }\left(t_{i}\right)\right)\; & & \;\\ & =\frac{{Ŝ}_{1}^{W}\left(t_{i-1}\right)\Delta N_{01}\left(t_{i+1}\right)}{Y_{0}^{*}\left(t_{i}\right)}\; & & \;\\ & ={Ŝ}_{1}^{W}\left(t_{i-1}\right)\left(1-\frac{\Delta N_{01}\left(t_{i}\right)}{Y_{0}^{*}\left(t_{i}\right)}\right)\frac{\Delta N_{01}\left(t_{i+1}\right)}{Y_{0}^{*}\left(t_{i+1}\right)}\; & & \;\\ & ={Ŝ}_{1}^{W}\left(t_{i-1}\right)\frac{\Delta N_{01}\left(t_{i}\right)}{Y_{0}^{*}\left(t_{i}\right)},\; & & \;\\ & \; & \end{array}$$

which completes the proof.

## Pre-publication history

The pre-publication history for this paper can be accessed here:

http://www.biomedcentral.com/1471-2288/11/144/prepub

## Supplementary Material

Additional file 1 **List of all participating hospitals in the CAPO data set used in this study**. Click here for file

Additional file 2 Supplement describing use of R for calculation of the cumulative incidence estimator. Click here for file

Additional file 3 **R code for the supplement**. Click here for file

Additional file 4 **Data for the supplement**. Click here for file

Additional file 5 **List of CAPO investigators and their affiliations**. Click here for file
