# Supplementary material for: How to handle mortality when investigating length of hospital stay and time to clinical stability
Source: BMC Med Res Methodol. 2011 Oct 26;11:144. doi: 10.1186/1471-2288-11-144 (PMC3269825; doi:10.1186/1471-2288-11-144)
Supplement: Additional file 1 — List of all participating hospitals in the CAPO data set used in this study. [file 1471-2288-11-144-S1.PDF]

List of all participating hospitals in the CAPO data set used in this study

ID	Name	City	State	Country	Region
1	University of Louisville	Louisville	KY	USA	USA/Canada
2	VA Medical Center	Louisville	KY	USA	USA/Canada
10	S. Maria della Misericordia	Udine	-	Italy	Europe
12	Hospital de Clinicas, Jose de San Martin	Buenos Aires	B.A	Argentina	Latin America
14	Hospital Universitario de Caracas	Caracas	Distrito Metropolitano	Venezuela	Latin America
16	Instituto de Neumonologia y Cirugia Toracica	Barcelona	-	Spain	Europe
17	Instituto Nacional del Torax	Santiago	-	Chile	Latin America
18	Hackensack University Medical Center	Hackensack	NJ	USA	USA/Canada
19	Policlinico	Milan	-	Italy	Europe
20	Ospedale L. Sacco	Milan	-	Italy	Europe
21	Hospital Dr Oscar Alende	Mar del Plata	Bs.As	Argentina	Latin America
22	University of Alberta Hospital	Edmonton	Alberta	Canada	USA/Canada
25	University of Santo Tomas Hospital	Manila	-	Philippines	Latin America
26	Instituto Medico Platense	La Plata	BsAs.	Argentina	Latin America
27	Hospital Universitario Joan XXIII de Tarragona	Tarragona	-	Spain	Europe
28	Hopital Maisonneuve-Rosemont	Montreal	QC	Canada	USA/Canada
29	National Kidney and Transplant Institute	Quezon City	Metro Manila	Philippines	Latin America
34	IRCCS Fondazione Policlinico	Milan	-	Italy	Europe
36	City Hosp. E.v.Behring/Lungenklinik Heckeshorn	Berlin	-	Germany	Europe
40	IDIM A. Lanari	Buenos Aires	.	Argentina	Latin America
43	Hospital Sant Pau i Santa Tecla	Tarragona	-	Spain	Europe
44	Northeastern Ohio Universities	Akron	OH	USA	USA/Canada
48	Hospital Nostra Senyora de Meritxell	Escaldes - Engordany	Andorra	Andorra	Latin America
52	Royal Alexandra Hospital	Edmonton	Alberta	Canada	USA/Canada
53	Sturgeon Community Hospital	St. Albert	AB	Canada	USA/Canada
54	Clinica las Condes	Santiago	.	Chile	Latin America
56	Hospital Español de La Plata	La Plata	Buenos Aires	Argentina	Latin America
57	Hospital Rodolfo Rossi	La Plata	Buenos Aires	Argentina	Latin America
59	Misericordia Hospital	Edmonton	AB	Canada	USA/Canada
60	Grey Nuns Hospital	Edmonton	AB	Canada	USA/Canada
61	Hospital Universitario La Fe	Valencia	Valencia	Spain	Europe
62	University of Texas Health Science Center	San Antonio	Texas	USA	USA/Canada
63	Pontificia Univesidad Católica de Chile	Santiago	Santiago	Chile	Latin America
64	Hospital Enrique Tornu	Buenos Aires	Bs As	Argentina	Latin America
65	Hospital Universitario Austral	Pilar	Buenos Aires	Argentina	Latin America
66	Hospital Francisco J. Muñiz	Buenos Aires	Buenos Aires	Argentina	Latin America

67	Sanatorio 9 de Julio	San Miguel de Tucuman	Tucuman	Argentina	Latin America
68	Clinica Uruguay	Concepcion del Uruguay	Entre Rios	Argentina	Latin America
71	Providence Hospital	Washington	DC	USA	USA/Canada
76	Profesor Bernardo Houssay	Vicente Lopez	Buenos Aires	Argentina	Latin America
