# Supplementary material for: How to handle mortality when investigating length of hospital stay and time to clinical stability
Source: BMC Med Res Methodol. 2011 Oct 26;11:144. doi: 10.1186/1471-2288-11-144 (PMC3269825; doi:10.1186/1471-2288-11-144)
Supplement: Additional file 2 — Supplement describing use of R for calculation of the cumulative incidence estimator. [file 1471-2288-11-144-S2.PDF]

R code for calculating the competing risks estimates

Guy Brock, Chris Barnes, Julio Ramirez, and John Myers
University of Louisville
Louisville, KY, USA 40202

August 18, 2011

Contents

1 Preliminaries **1**

2 Cumulative Incidence Function **3**

1 Preliminaries

This supplement illustrates the R code used to calculate the competing risks estimates of the cumulative incidence function and conditional probability function for hospital discharge. The data used for the example is contained in the comma-separated file "SupplementData.csv", and the R code to reproduce the analysis detailed here is in the file "CompRiskSupp.R". To begin, you need to install the R statistical software program, which can be obtained from http://www.r-project.org (**?**). Once the software is installed, open the program and install the `cmprsk` package (Gray, 2011). This can be done by clicking "Packages" and then "Install Package(s)..." from the top menu. Select an appropriate CRAN mirror, then select the `cmprsk` package from the subsequent window.

Next, navigate to the directory where you downloaded the data file "SupplementData.csv" and R code "CompRiskSupp.R" by selecting "File" and then "Change dir..." from the top menu. Open the R script "CompRiskSupp.R" by selecting "File" and then "Open script..." from the top menu. The program should open the script containing the R code which accompanies this file. The R code can be copied and pasted directly into the "R Console" (the command prompt), and will reproduce the analysis

presented here. Alternatively, users can type all the commands following the *R>* prompt below at the R Console.

The first step is to load the data file and store it in an object called `Data`, using the following command (The names of all objects used to store data and results are not fixed and can be changed by the user):

```
R> Data <- read.csv("SupplementData.csv", header = TRUE)
```

The data file contains three variables, `LOS`, `CR.event`, and `RiskClass`. Also, the total number of subjects in the data file is 1,232. The information can be seen using the following commands:

```
R> names(Data)

[1] "LOS"       "CR.event"  "RiskClass"

R> nrow(Data)

[1] 1232
```

The `LOS` variable contains the length of hospital stay for each patient, up to 30 days. Patients with hospital stays longer than 30 days were censored at 30 days. The `CR.event` variable contains information on the event type for each patient: a '1' indicates discharge from the hospital, a '2' indicates the patient died in the hospital, and a '0' indicates that the information was censored. The `RiskClass` variable indicates the pneumonia severity index (PSI) risk class for each patient. Here, we are restricting analysis to patients in risk classes IV and V.

A total of 184 patients died within the first 30 days of admittance to the hospital (no patients died after the first 30 days). The number of patients who were discharged, died, or censored can be obtained using the `table()` function:

```
R> table(Data$CR.event)

  0   1   2
 56 992 184
```

2 Cumulative Incidence Function

Next, we detail how to obtain the estimate of the cumulative incidence function for the marginal probability of hospital discharge and in-hospital mortality. The use of competing risks are appropriate in this case, since patient mortality prevents subsequent discharge of that patient. Also, hospital discharge prevents the future occurrence of mortality *within the hospital*. First, the `cmprisk` package needs to be loaded into the current R session, using the following command:

```
R> library(cmprsk)
```

Estimates of the cumulative incidence function are obtained using the `cuminc()` function. The function takes arguments `ftime` for the 'failure' time (here, time to discharge or in-hospital mortality) and `fstatus` to indicate the type of outcome. A detailed help page for the `cuminc()` function is obtained by typing `?cuminc` or `help(cuminc)` at the R command prompt. We start by getting an overall estimate, which combines risk classes IV and V. The results are stored in an object called `CI.overall`.

```
R> CI.overall <- cuminc(ftime = Data$LOS, fstatus = Data$CR.event)
R> CI.overall

Estimates and Variances:
$est
             5        10        15        20        25
1 1 0.23457792 0.5243506 0.6745130 0.7459416 0.7784091
1 2 0.06087662 0.1055195 0.1282468 0.1420455 0.1485390

$var
                5           10           15           20           25
1 1 1.454135e-04 2.004294e-04 1.750319e-04 1.504044e-04 0.0001366185
1 2 4.637362e-05 7.615595e-05 8.980497e-05 9.752167e-05 0.0001010098
```

The printed result shows the estimated marginal probability of each outcome (1=discharge, 2=death) at days 5, 10, 15, 20, and 25, along with the variance for each estimate. For example, the estimated marginal probability of discharge by day 10 is 52.4%, and the estimated marginal probability of death by day 10 is 10.6%. The information can be plotted to get an overall picture of the cumulative incidence of each event over the entire time course, given in Figure 1.

```
R> plot(CI.overall, curvlab = c("Discharge", "Death"), xlab = "Days")
```

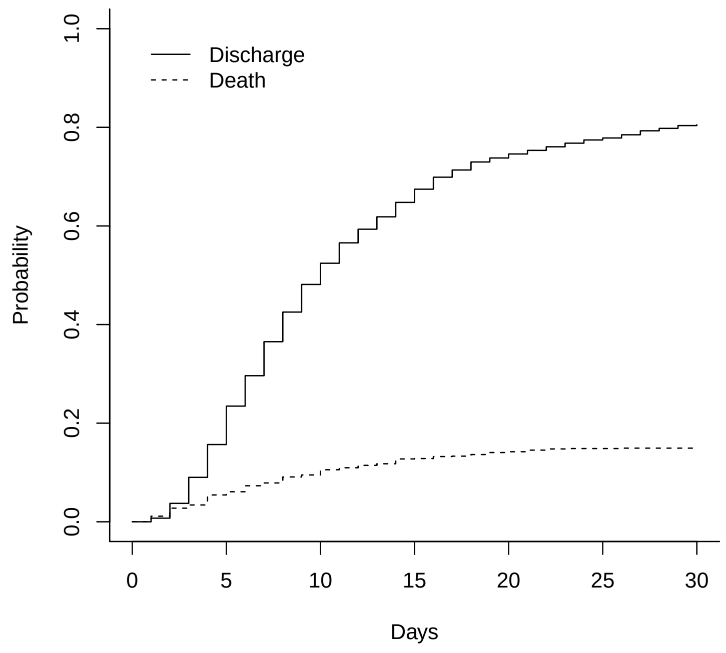

Figure 1: Plot of the cumulative incidence for hospital discharge and death, elderly patients in RC IV and V hospitalized with community-acquired pnuemonia.

Next, we obtain seperate estimates of the cumulative incidence for hospital discharge and in-hospital mortality for PSI risk classes IV and V. This is accomplished by using the `group` argument in the `cmprsk()` function, and the result is stored in the object `CI.4vs5`.

```
R> CI.4vs5 <- cuminc(ftime = Data$LOS, fstatus = Data$CR.event,
+     group = Data$RiskClass)
```

The result can again be plotted, this time with separate curves for each risk class (Figure 2).

```
R> plot(CI.4vs5, lty = c(1, 1, 2, 2), col = c("black", "blue", "black",
+     "blue"), curvlab = c("Discharge, RC IV", "Discharge, RC V",
+     "Death, RC IV", "Death, RC V"), xlab = "Days")
```

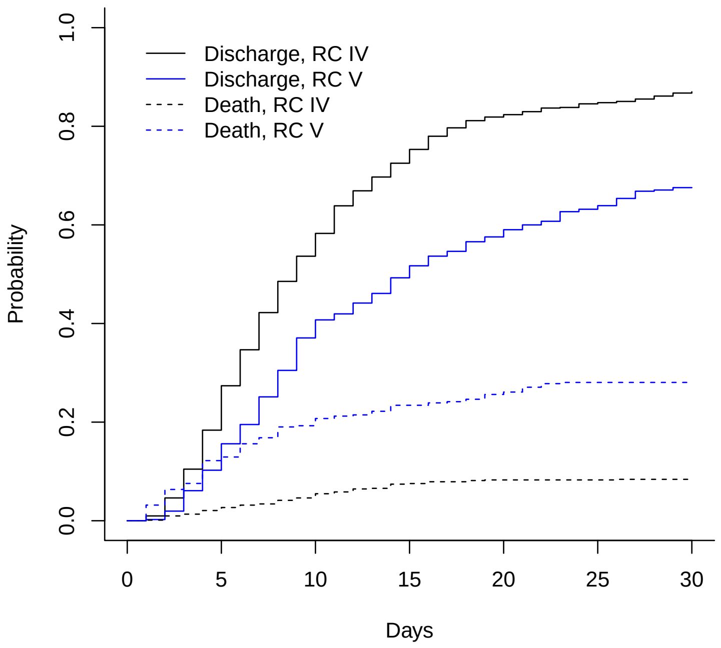

Figure 2: Plot of the cumulative incidence for hospital discharge and death, by PSI risk class IV and V.

The incidence of death is higher in risk class V vs IV, and the incidence of discharge is higher for risk class IV. A formal test for differences in length of stay and in-hospital mortality between PSI risk classes IV and V can be performed using the modified χ^2 statistic outlined in (Gray, 1988).

```
R> CI.4vs5$Tests
```

```
      stat pv df
1 70.33101  0  1
2 86.41816  0  1
```

The χ^2 statistic for differences in incidence of hospital discharge (outcome '1') is 70.33, with a p-value of 'zero' (less than 10^{-16}). Since we are comparing two groups, there is a single degree of freedom (df) for the test. Similarly, the χ^2 statistic for differences in incidence of in-hospital mortality (outcome '1') is 86.42, with a p-value of essentially zero on one degree of freedom.

Further information on using R to perform competing risks analysis can be found in (Scrucca et al., 2007). The *cmprsk* package does have several limitations in that it can only handle right-censored data and it uses an estimate of the variance which has been shown to be suboptimal Braun and Yuan (2007). Alternative `R` packages which can handle left-truncated data and use the Greenwood-type estimate of the variance include *etm* Allignol et al. (2011), *msSurv* Ferguson et al. (2011), and *mstate* de Wreede et al. (2011). In particular, the *mstate* package has a `Cuminc` function which is very similar to the `cuminc` function in *cmprsk*. However, only the *cmprsk* package includes test statsitics Gray (1988) for comparing cumulative incidence curves.

References

A. Allignol, M. Schumacher, and J. Beyersmann. Empirical transition matrix of multi-state models: the *etm* package. *J Stat Softw*, 38, 2011.

T. M. Braun and Z. Yuan. Comparing the small sample performance of several variance estimators under competing risks. *Stat Med*, 26(5):1170–80, 2007.

L. C. de Wreede, M. Fiocco, and H. Putter. *mstate*: an R package for the analysis of competing risks and multi-state models. *J Stat Softw*, 38, 2011.

N. Ferguson, G. Brock, and S. Datta. *msSurv: Nonparametric estimation for multi-state models*, 2011. URL `cran.r-project.org/web/packages/msSurv/msSurv.pdf`. R package version 1.0-2.

R. Gray. A class of k-sample tests for comparing the cumulative incidence of a competing risk. *Ann Stat*, 16:1141–1154, 1988.

R. Gray. *cmprsk: Subdistribution Analysis of Competing Risks*, 2011. URL `cran.r-project.org/web/packages/cmprsk/index.html`. R package version 2.2-2.

L. Scrucca, A. Santucci, and F. Aversa. Competing risk analysis using r: an easy guide for clinicians. *Bone Marrow Transplant*, 40(4):381–7, 2007.
