# Supplementary material for: How to handle mortality when investigating length of hospital stay and time to clinical stability
Source: BMC Med Res Methodol. 2011 Oct 26;11:144. doi: 10.1186/1471-2288-11-144 (PMC3269825; doi:10.1186/1471-2288-11-144)
Supplement: Additional file 5 — List of CAPO investigators and their affiliations. [file 1471-2288-11-144-S5.PDF]

CAPO investigators and their affiliations

The authors gratefully acknowledge the work of the CAPO investigators in building and maintaining the CAPO data set. The CAPO investigators are:

Dr. Marty Allen, University of Louisville, KY, USA; Dr. Raul Nakamatsu, Veterans Affairs Medical Center, Louisville, KY, USA; Dr. Jose Bordon, Providence Hospital, Washington, D.C., USA; Dr. Thomas File, Jr., Summa Health System, Akron, OH, USA; Dr. Peter Gross, Hackensack University Medical Center, Hackensack, NJ, USA; Dr. Thomas Marrie, University of Alberta Hospital, Sturgeon Community Hospital, Grey Nuns Hospital, and Royal Alexandra Hospital, Edmonton, Alberta, Canada; Dr. Karl Weiss, Maisonneuve-Rosemont Hospital, University of Montreal, Montreal, Canada; Dr. Francesco Blasi, Instituto Malattie Respirattorio, University of Milan, Instituto di Recerca e Cura a Carattere Scientifico, Policlinico, Milan, Italy; Dr. Roberto Cosentini, Policlinico, Milan, Italy; Dr. Delfino Legnani, Ospedale L. Sacco, Milan, Italy; Dr. Antoni Torres, Instituto de Neumonologia y Cirugia Toracica, CIBER Enfermedades Respiratorias, Barcelona, Spain; Dr. Maria Bodi, Hospital Universitario Joan XXIII, Tarragona, Spain; Dr. Jose Porras, Hospital Sant Pau i Santa Tecla , Tarragona, Spain; Dr. Jordi Rello, Critical Care Department, Joan XXIII University Hospital, CIBER Enfermedades Respiratorias, University Rovira & Virgili, Tarragona, Spain; Dr. Rosario Menendez, Pneumology Service, Hospital Universitario La Fe; CIBER Enfermedades Respiratorias, Valencia, Spain; Dr. Harmut Lode, City Hosp. E.v.Behring/Lungenklinik Heckeshorn, Berlin, Germany; Dr. Jorge Roig, Hospital Nostra Senyora de Meritxell, Escaldes, Andorra; Dr. Guillermo Benchetrit, IDIM A. Lanari, Buenos Aires, Argentina; Dr. Jorge Corral, Hospital Dr Oscar Alende, Mar del Plata, Argentina; Dr. Jose Gonzalez, Hospital Enrique Tornu, Buenos Aires, Argentina; Dr. Lautaro de Vedia, Hospital Francisco J. Muniz, Buenos Aires, Argentina; Dr. Gustavo Lopardo, Profesor Bernardo Houssay, Buenos Aires, Argentina; Dr. Carlos Luna, Hospital de Clinicas, Buenos Aires, Argentina; Dr. Jorge Martinez, Instituto Medico Platense, La Plata, Argentina; Dr. Lucia Marzoratti, Sanatorio 9 de Julio, Tucuman, Argentina; Dr. Maria Rodriguez, Hospital Rodolfo Rossi, La Plata, Argentina; Dr. Alejandro Videla, Hospital Universitario Austral, Buenos Aires, Argentina; Dr. Federico Arteta, Hospital Luis Gomez Lopez-Ascardio, Barquisimeto, Venezuela; Dr. Gur Levy, Hospital Universitario de Caracas, Caracas, Venezuela; Patricia Fernandez, Instituto Nacional del Torax, Santiago, Chile; Dr. Maria Parada, Clinica las Condes, Santiago, Chile; Dr. Juan Manuel Luna, Hospital Nacional Roosevelt, Guatemala; Dr. Marilyn Mateo, University of Santo Thomas Hospital, Manila, Philippines; Dr. Myrna Mendoza, University of the Philippines and National Kidney and Transplant Institute, Manila, Philippines; Dr. Charles Feldman, Johannesburg Hospital, Johannesburg, South Africa.
